# Pilot Study to Establish a Novel Five-Gene Biomarker Panel for Predicting Lymph Node Metastasis in Patients With Early Stage Endometrial Cancer

**DOI:** 10.3389/fonc.2019.01508

**Published:** 2020-01-21

**Authors:** Chia-Yen Huang, Kuang-Wen Liao, Chih-Hung Chou, Sirjana Shrestha, Chi-Dung Yang, Men-Yee Chiew, Hsin-Tzu Huang, Hsiao-Chin Hong, Shih-Hung Huang, Tzu-Hao Chang, Hsien-Da Huang

**Affiliations:** ^1^Department of Biological Science and Technology, National Chiao Tung University, Hsinchu, Taiwan; ^2^Department of Obstetrics and Gynecology, Gynecologic Cancer Center, Cathay General Hospital, Taipei, Taiwan; ^3^School of Medicine, Fu Jen Catholic University, New Taipei City, Taiwan; ^4^Institute of Molecular Medicine and Bioengineering, National Chiao Tung University, Hsinchu, Taiwan; ^5^Center for Intelligent Drug Systems and Smart Bio-devices (IDS2B), National Chiao Tung University, Hsinchu, Taiwan; ^6^Institute of Bioinformatics and Systems Biology, National Chiao Tung University, Hsinchu, Taiwan; ^7^School of Life and Health Sciences, Chinese University of Hong Kong, Shenzhen, China; ^8^Warshel Institute for Computational Biology, Chinese University of Hong Kong, Shenzhen, China; ^9^Department of Pathology, Cathay General Hospital, Taipei, Taiwan; ^10^International Center for Health Information Technology, Taipei Medical University, Taipei, Taiwan; ^11^Graduate Institute of Biomedical Informatics, College of Medical Science and Technology, Taipei Medical University, Taipei, Taiwan

**Keywords:** endometrial cancer, lymph node metastasis, RNA sequencing, TCGA, prediction model

## Abstract

**Introduction:** In the United States and Europe, endometrial endometrioid carcinoma (EEC) is the most prevalent gynecologic malignancy. Lymph node metastasis (LNM) is the key determinant of the prognosis and treatment of EEC. A biomarker that predicts LNM in patients with EEC would be beneficial, enabling individualized treatment. Current preoperative assessment of LNM in EEC is not sufficiently accurate to predict LNM and prevent overtreatment. This pilot study established a biomarker signature for the prediction of LNM in early stage EEC.

**Methods:** We performed RNA sequencing in 24 clinically early stage (T1) EEC tumors (lymph nodes positive and negative in 6 and 18, respectively) from Cathay General Hospital and analyzed the RNA sequencing data of 289 patients with EEC from The Cancer Genome Atlas (lymph node positive and negative in 33 and 256, respectively). We analyzed clinical data including tumor grade, depth of tumor invasion, and age to construct a sequencing-based prediction model using machine learning. For validation, we used another independent cohort of early stage EEC samples (*n* = 72) and performed quantitative real-time polymerase chain reaction (qRT-PCR). Finally, a PCR-based prediction model and risk score formula were established.

**Results:** Eight genes (*ASRGL1, ESR1, EYA2, MSX1, RHEX, SCGB2A1, SOX17*, and *STX18*) plus one clinical parameter (depth of myometrial invasion) were identified for use in a sequencing-based prediction model. After qRT-PCR validation, five genes (*ASRGL1, RHEX, SCGB2A1, SOX17*, and *STX18*) were identified as predictive biomarkers. Receiver operating characteristic curve analysis revealed that these five genes can predict LNM. Combined use of these five genes resulted in higher diagnostic accuracy than use of any single gene, with an area under the curve of 0.898, sensitivity of 88.9%, and specificity of 84.1%. The accuracy, negative, and positive predictive values were 84.7, 98.1, and 44.4%, respectively.

**Conclusion:** We developed a five-gene biomarker panel associated with LNM in early stage EEC. These five genes may represent novel targets for further mechanistic study. Our results, after corroboration by a prospective study, may have useful clinical implications and prevent unnecessary elective lymph node dissection while not adversely affecting the outcome of treatment for early stage EEC.

## Introduction

As the gynecologic malignancy with the highest prevalence in Western countries (1) and Taiwan (2), endometrial cancer has become more common in recent years (3, 4). Endometrial endometrioid carcinoma (EEC) is the most prevalent histological type of endometrial cancer (5). The primary treatment for patients with medically operable EEC is surgical staging, which involves collecting peritoneal fluid for cytologic examination; inspecting the whole abdominal and pelvic cavities, after which any suspicious lesions are biopsied or excised; total extrafascial hysterectomy along with bilateral salpingo-oophorectomy; and lymph node evaluation (6). This procedure obtains complete pathologic and prognostic information for tailoring further adjuvant therapy.

The surgical treatment employed in patients with endometrial cancer has changed. Previously, lymph node assessment in EEC comprised dissection of the para-aortic and pelvic nodes in all patients (6). However, EEG is often diagnosed early, when the risk of lymph node metastasis (LNM) is low (5). In clinical stage I EEC, the incidence of pelvic and para-aortic nodal metastases is only 10 and 6%, respectively (7). Most patients with early stage EEC can avoid lymph node dissection to prevent morbidities. Nerve injury, prolonged operation time, lymphedema, blood loss, and lymphocyst formation are the principal morbidities associated with lymph node dissection (7–10). Thus, a more selective lymphadenectomy is suggested for patients with EEC to prevent overtreatment (11).

Sentinel lymph node (SLN) mapping can be an alternative to full lymphadenectomy in patients with EEC. According to the National Comprehensive Cancer Network guidelines (12), SLN mapping can identify metastatic lymph nodes in diseases confined to the uterus (13–16). SLN mapping has improved surgeons' detection of LNM in small-volume disease and has considerably reduced intraoperative and postoperative morbidity. However, the skills, knowledge, and attention to technical detail of the surgeon are crucial. Only institutions with the relevant expertise in SLN mapping should perform the procedure to minimize false negatives, which may compromise treatment outcomes (12). Most critically, SLN mapping is performed during surgery and is not a preoperative setting for the assessment of lymph node status.

At present, lymph node status is preoperatively assessed using clinical risk factors including tumor invasion depth, tumor grade, or tumor size, but in clinical practice, such assessment has a low positive predictive value (17–19). Scholars have concluded that image-based diagnostic tools—including computed tomography (20), magnetic resonance imaging (21, 22), and positron emission tomography (23)—are inadequate for detection of micrometastatic nodal disease because they have low sensitivity. Recent advances in molecular profiling have provided important insights into the biological nature of tumors. These advances have enabled researchers to investigate gene expression signatures as diagnostic tools for clinical decision-making (24) and prediction of outcomes, including tumor metastases (25–27).

In the current study, we investigated the clinicopathological parameters and molecular markers of LNM in patients with early clinical stage EEC by analyzing gene expression in surgical specimens from such patients. Such genetic signatures may help guide individualized therapies and reduce the surgical morbidities of patients with EEC.

## Methods

### Cathay General Hospital Dataset

#### Patient Enrollment and Sampling

The Institutional Review Board of Cathay General Hospital (CGH) approved the experimental protocols employed in this study. Prior to surgery, informed consent was obtained from all participants (IRB: CGH-P104102). Between March 2011 and December 2017, 113 patients with EEC who underwent staging surgery through either laparotomy or a minimally invasive procedure were enrolled. We included patients with (i) unambiguous histologic diagnosis of endometrioid carcinoma and absence of a mixed tumor type, (ii) tissue that was sufficient to enable RNA extraction, and (iii) no prior treatment history. Clinical TMN staging before surgery was evaluated according to the *American Joint Committee on Cancer Staging Manual, Seventh Edition* (28). Only patients receiving complete surgical staging surgery—that is, undergoing total hysterectomy and/or bilateral salpingo-oophorectomy, para-aortic lymphadenectomy, and bilateral pelvic lymph node dissection—were included. Our aim was to identify the molecular signature of early clinical stage EEC. Therefore, only patients with clinical T1 tumors documented through preoperative magnetic resonance imaging were included.

**Figure 1:**
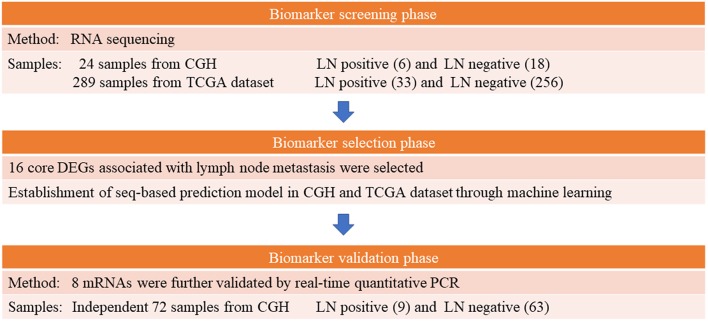
Schematic of study workflow.

Following hysterectomy, tissue specimens were collected and preserved in RNAlater® (Qiagen, Valencia, CA, USA) immediately for storage at −80°C until they were analyzed. The 2009 International Federation of Obstetrics and Gynecologic staging system was the basis of our EEC tumor staging and grading criteria (29). A gynecological pathologist (S-HH) reviewed the histopathology. Lymph node status was considered positive if metastasis was histologically proven and negative if no metastasis was detected in the surgical specimen and during at least the subsequent 1 year. Clinical data collected prior to hysterectomy regarding age, menopausal status, and body mass index were recorded. We examined the patients' detailed medical records for the period up to December 31, 2018. A multistep case–control study was designed to identify mRNA markers for predicting LNM in patients with EEC. Figure 1 presents a study workflow schematic. Additionally, we applied the REMARK reporting recommendations for prognostic biomarkers (Supplement Table 1) (30).

#### RNA Sequencing and Data Processing

In the initial screening phase, we selected 6 patients with and 18 patients without LNM for RNA sequencing (Table 1). For RNA preparation, ~25 mg of tissue from an EEC tumor specimen underwent homogenization in a TissueLyser II bead mill (Qiagen) running at 30 Hz for 10–20 s and chilled lysis buffer from the AllPrep RNA/DNA/Protein Mini Kit (Qiagen) in accordance with the guidelines of the manufacturer. Quantification of the RNA elution was performed using a Nanodrop spectrophotometer (Thermo Fisher Scientific, MA, USA), and the elution was further examined using a Bioanalyzer 2200 (Agilent Technologies, Santa Clara, CA, USA), which output an RNA integrity number between 1 (indicating lowest quality and most degradation) and 10 (indicating highest quality and least degradation) (31). Samples with an RNA integrity number <7—which may have substantially affected the results of sequencing (e.g., 3′-5′ transcript bias and uneven gene coverage) and led to inaccurate conclusions—were not included in the analysis. A TruSeq mRNA Sample Preparation Kit V2 (Illumina, San Diego, CA, USA) used according to the manufacturer protocol was employed to construct RNA sequencing libraries. Briefly, the protocol for preparing these RNA sequencing libraries involved poly-A RNA isolation, RNA fragmentation, reverse transcription to cDNA using random primers, 3′-end adenylation, adapter ligation, and polymerase chain reaction (PCR) enrichment. The resultant cDNA libraries quantified through quantitative real-time (qRT)-PCR (using the Roche LightCycler® 480 System) and Qubit fluorometry (Invitrogen, Carlsbad, CA, USA). After they were purified, quantified, and validated, the libraries were sequenced on the Illumina Sequencing System (Next-seq 500 sequencing system, 75 single-end) according to the manufacturer's standard workflow. The RNA sequencing data were subject to quadripartite analysis involving (i) quality control, (ii) alignment to the reference genome, (iii) splice junction discovery and transcript assembly, and (iv) abundance estimation and normalization. We deposited the raw sequences of the EEC samples in the National Center for Biotechnology Information Sequence Read Archive (access number PRJNA554534).

**Table 1:** Basic characteristics of patients from CGH for RNA sequencing (CGH dataset, *n* = 24).

**Variable**	**Lymph node positive** **(*n* = 6)**	**Lymph node negative** **(*n* = 18)**	***p***
Age, years (median)	61.8	54.8	0.73^a^
Body mass index (mean)	23.7	24.2	0.55^b^
Menopausal status, *n* (%)			1.00^c^
Premenopausal	1 (16.7%)	3 (16.7%)	
Perimenopausal	5 (83.3%)	15 (83.3%)	
Surgical approach, *n* (%)			1.00^c^
Open	5 (83.3%)	13 (72.2%)	
Minimally invasive	1 (16.6%)	5 (27.8%)	
Tumor grade			0.007^c^
1	1 (16.7%)	15 (83.3%)	
2	3 (50%)	2 (11.1%)	
3	2 (33.3%)	1 (5.6%)	
Myometrial invasion			0.061^c^
<1/2	1 (16.7%)	12 (66.7%)	
≥1/2	5 (83.3%)	6 (33.3%)	
Lymphovascular space invasion			<0.0001^c^
Present	6 (100%)	5 (27.8%)	
Absent	0 (0%)	13 (72.2%)	

a t-test.

b Mann–Whitney U-test.

c *Fisher-exact test*.

Our ultimate goal was to identify clinically predictive markers by using RT-PCR to measure specific gene expression in surgical specimens of patients with EEC. We validated the differentially expressed genes (DEGs) identified from next-generation sequencing data by using RT-PCR. Therefore, the selection of the reference gene for comparison was critical. The interpretation of RNA sequencing results is highly dependent upon the reference genes employed for normalization (32). To identify a gene that was highly expressed in both The Cancer Genome Atlas (TCGA; www.cancergenome.nih.gov) and CGH datasets with minimal variability between samples, NormFinder (https://moma.dk/normfinder-software) was used for identification of the appropriate normalization gene (33). Using NormFinder, candidate normalization genes could be ranked by the stability of their expression in the CGH and TCGA datasets. *RPL19* was identified as a suitable reference gene in this study.

### TCGA Dataset

#### Databases

We analyzed mRNA expression profiles among patients present in TCGA uterine corpus endometrial carcinoma series. Data were collected in August 2016. Only cases involving a histologic EEC diagnosis and with complete clinical information regarding tumor grade, lymph node status, and myometrial invasion depth were selected for analysis. Additionally, we selected only patients with clinical stage I (negative lymph nodes) or IIIC (positive lymph nodes) disease for comparison. After filtering out excluded cases, 289 patients with endometrioid carcinoma were selected for analyses (Table 2). TCGA comprises three levels of data. We downloaded level 3 RNAseqV2 normalized data, containing gene expression information, for the aforementioned 289 patients. The patients and their clinical data were merged into a single dataset to eliminate duplicate entries.

**Table 2:** Basic characteristics of TCGA patients with uterine corpus endometrial carcinoma (TCGA dataset, *n* = 289).

**Variable**	**Lymph node positive** **(*n* = 33)**	**Lymph node negative** **(*n* = 256)**	***p***
Age, years (mean)	59.4	63.0	0.69^a^
Body mass index (mean)	33.1	34.8	0.46^b^
Menopausal status, *n* (%)			
Premenopausal	4 (12.1%)	19 (7.4%)	0.58^c^
Perimenopausal	2 (6.1%)	10 (3.9%)	
Postmenopausal	26 (78.8%)	210 (82.0%)	
Indeterminate & NA	1 (3.0%)	17 (6.6%)	
Surgical approach, *n* (%)			0.005^c^
Open	27 (82.8%)	142 (55.5%)	
Minimally invasive	6 (18.2%)	109 (42.6%)	
NA		5 (1.9%)	
Tumor grade, *n* (%)			
1	2 (6.1%)	78 (30.5%)	0.001^c^
2	8 (24.2%)	79 (30.9%)	
3	23 (69.7%)	99 (38.7%)	
Myometrial invasion, *n* (%)			<0.0001^c^
<1/2	9 (27.3%)	165 (64.5%)	
≥1/2	24 (72.7%)	91 (35.5%)	

a t-test.

b Mann–Whitney U-test.

c *Chi-square test*.

#### RNA Sequence Data Processing and Differential Expression Analysis

We corrected the raw data downloaded from TCGA for background and normalization. The expression level was calculated using the Bioconductor package. After the raw data were preprocessed and normalized to the *RPL19* reference gene, the DEGs between patients with and without LNM were identified. The DEGs were screened using the cut-off criteria, which were set as a fold change (FC) ≧ 1.5 and an adjusted Wilcoxon rank-sum *p*-value of <0.05.

### Sequencing-Based Model Construction and Feature Selection Through Machine Learning

The DEGs in both the CGH and TCGA datasets were selected as core DEGs. The prediction model was tuned using variables including tumor grade, myometrial invasion depth, and age along with gene expression data. Although, lymphovascular space invasion (LVSI) was previously considered a crucial determinant of LNM (34), LVSI information is unavailable in TCGA. In addition, the LVSI status is not routinely reported in small presurgical biopsy specimens collected through dilatation and curettage or endometrial biopsy; therefore, we did not include LVSI in our model. Four machine learning methods—namely, the random forest (35), sequential minimal optimization, J48, and Naïve Bayes methods—were employed for predictions. Threefold cross validation with 10 repetitions was used to assess these methods' performance.

High sensitivity was critical to this study's model. However, the CGH and TCGA datasets have imbalanced numbers of patients with and without LNM. Therefore, we employed the WEKA cost-sensitive classifier to enhance the penalty for false-negative samples. Finally, the predictive performance of the sequencing-based model with the CGH and TCGA datasets was examined.

### qRT-PCR Validation and PCR-Based Prediction Model Construction Through Machine Learning

To validate our findings, we performed qRT-PCR on clinical EEC tissues from another independent set of patients with early stage EEC (Table 3). The mRNA expression levels of eight genes—*ASRGL1, ESR1, EYA2, MSX1, RHEX, SCGB2A1, SOX17*, and *STX18*—were evaluated using TaqMan qRT-PCR in triplicate. We performed qRT-PCR following a previous study (36). Briefly, for amplification, we used 2 μL of cDNA at 1:10 dilution and TaqMan real-time probes and primers (Applied Biosystems, Foster City, CA, USA). Supplement Table 2 lists the primer sequences. TaqMan Universal PCR Master Mix (ABgene, Rochester, NY, USA) and an ABI Prism 7900-HT Sequence Detection System (Applied Biosystems) were employed for all PCR. For each sample, individual genes' relative mRNA expression levels were normalized to *RPL19* expression levels through the comparative Ct (2^−ΔΔCt^) method (36). Five DEGs in the validation cohort were used for further machine learning and prediction model construction. The four machine learning methods mentioned in the previous subsection were employed to construct a PCR-based prediction model.

**Table 3:** Basic characteristics of validation cohort (*n* = 72).

**Variable**	**Lymph node positive** **(*n* = 9)**	**Lymph node negative** **(*n* = 63)**	***p***
Age, years (mean)	58.7	54.0	0.45^a^
Body mass index (mean)	24.3	25.6	0.18^b^
Menopausal status, *n* (%)			
Premenopausal	2 (22.2%)	18 (28.6%)	1.00^c^
Perimenopausal	7 (77.8%)	45 (71.4%)	
Surgical approach, *n* (%)			1.00^c^
Open	2 (22.2%)	17 (27.0%)	
Minimally invasive	7 (77.8%)	46 (73.0%)	
Tumor grade			0.0001^c^
1	1 (11.1%)	50 (79.4%)	
2	4 (44.4%)	8 (12.7%)	
3	4 (44.4%)	5 (7.9%)	
Myometrial invasion			0.023^c^
Inner 1/2	2 (22.2%)	42 (66.7%)	
Outer 1/2	7 (77.8%)	21 (33.3%)	
Number of lymph nodes removed			0.08^b^
Median (range)	26 (14–36)	17 (11–57)	
Lymphocascular space invasion			<0.0001^c^
Present	9 (100%)	17 (27.0%)	
Absent	0 (0%)	46 (73.0%)	

a t-test.

b Mann–Whitney U-test.

c *Fisher-exact test*.

### Risk Score Calculation and Predictive Model Construction

For clinical application of the mRNA signature, we used binary logistic regression analysis to generate a comprehensive set of multi-mRNA markers (combination of five mRNA markers) for the prediction of lymph node status. Subsequently, the expression levels of the mRNA markers were linearly combined into an LNM risk score as follows. Risk score = 0.249 × (Ct of *ASRGL1* – Ct of *RPL19*) + 0.166 × (Ct of *RHEX* – Ct of *RPL19*) + 0.434 × (Ct of *SCGB2A1* – Ct of *RPL19*) + 0.02 × (Ct of *SOX17* – Ct of *RPL19*) – 0.51 × (Ct of *STX18* – Ct of *RPL19*) – 4.06, where Ct is the threshold cycle of each gene. Area under the receiver operating characteristic (ROC) curve (AUC) for each (or the combined) mRNA marker was calculated using a 95% confidence interval to evaluate the performance of the mRNA expression markers in identifying patients with LNM. The optimal cutoffs and related specificity, sensitivity, and positive and negative predictive value (PPV and NPV) from ROC curves were determined using a common method. We considered *p* < 0.05 statistically significant.

### Statistical Analysis

We performed statistical analysis in SPSS version 15.0 (SPSS Inc., Chicago, IL, USA) for Windows. Intergroup differences in continuous variables were evaluated through unpaired *t* or Mann–Whitney *U* tests after data normality was confirmed using the Shapiro–Wilk test, and differences in categorical variables were evaluated through Fisher-exact or chi-square tests. For gene expression analysis, the Bioconductor package (version 3.6) in R (version 3.4.3) was used to identify DEGs between patients with and without LNM (37). The DEGs were screened using the cut-off criteria, which were set as FC ≧ 2 and adjusted Wilcoxon rank-sum *p* < 0.05 in CGH dataset. We employed R packages to statistically analyze the RNA sequencing and clinical data.

## Results

### Study Population's Basic Characteristics

The basic clinicopathological parameters of the patients in the CGH dataset (*n* = 24) are listed in Table 1. These patients were divided into those with negative [LN [–]; 18 patients [75%]] or positive lymph nodes [LN [+]; 6 patients [25%]]. These two groups were similar in body mass index, age, surgical route, and menopausal status. The LN (+) group had higher proportions of high-grade tumors (*p* = 0.007) and positive LVSI status (*p* < 0.0001).

From TCGA data, we identified 289 EEC patients with comprehensive lymph node status, genomic expression, and clinical data. Of these 289 patients, 256 (88.6%) were LN (–) and 33 (11.4%) were LN (+) (Table 2). Patients in TCGA with LNM were more likely to have undergone an open procedure (*p* = 0.005), a high-grade tumor (*p* = 0.001), or deep myometrial invasion (*p* < 0.0001; Table 2).

### DEGs Between Node-Positive and Node-Negative EEC in the CGH and TCGA Datasets

We conducted RNA sequencing for 24 patients in the CGH dataset. On average, we obtained 20.8 million reads from each of these 24 libraries. We assessed the quality of the reads by using FastQC, and all reads were discovered to have a Phred score higher than 30. Supplement Table 3 presents alignment and mapping quality metrics produced by Qualimap over the output BAM files through STAR. We discovered 779 DEGs between the LN (+) and LN (–) cancer tissues in the CGH dataset (FC ≧ 2 and *p* < 0.05 after adjustment for false discovery rate). Additionally, data on mRNA expression in patients with EEC were obtained from TCGA and included 33 LN (+) and 256 LN (–) EEC tissues. Between these tissues, 41 DEGs were identified (FC ≧ 1.5 and *p* < 0.05 after adjustment for false discovery rate).

### Sequencing-Based Model Construction and Feature Selection

**Figure 2:**
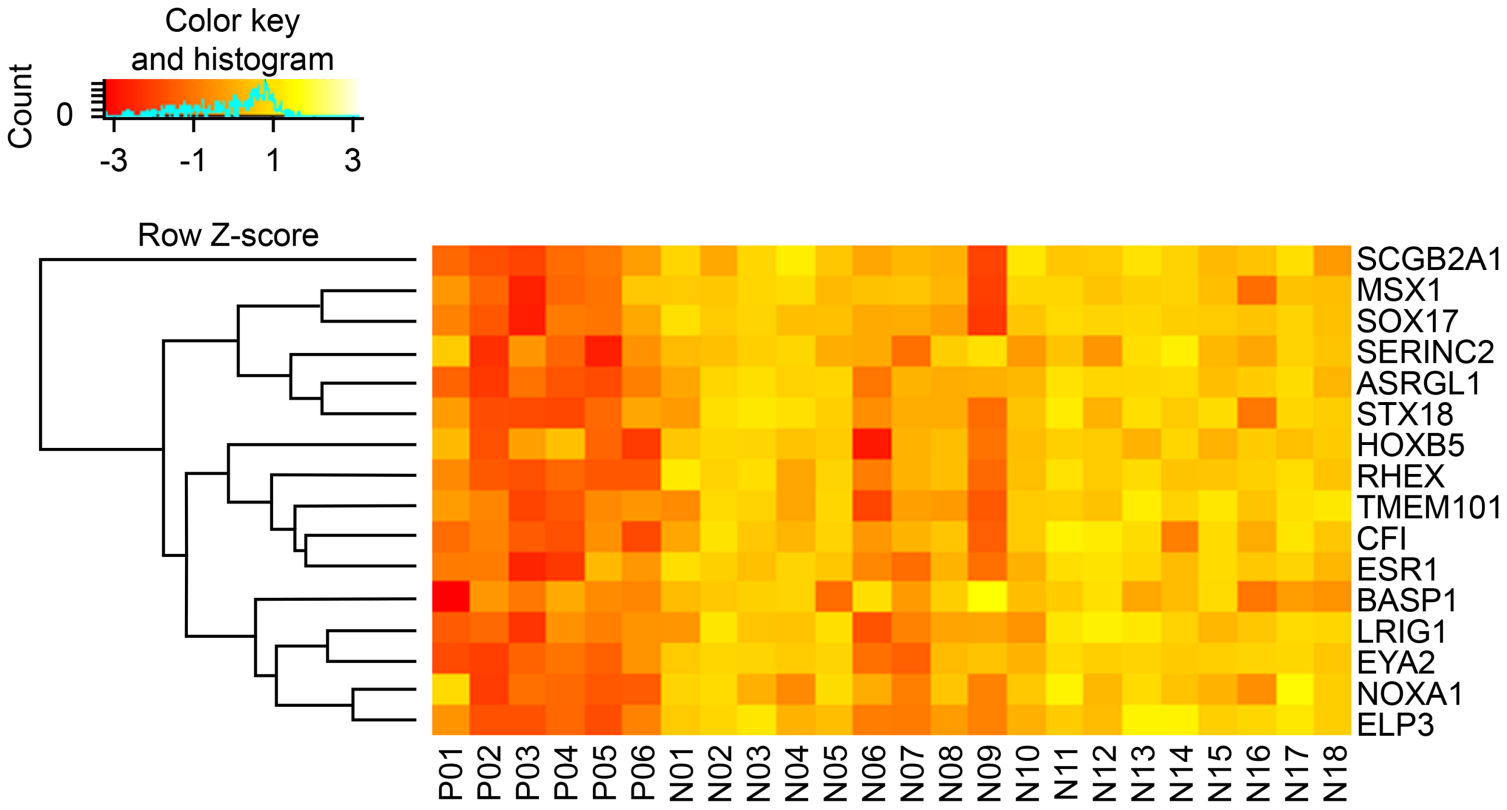
Hierarchical clustering heatmap of DEGs (rows) between LN (+) (P01–P06) and LN (–) (N01–N18) tumors. Yellow indicates high expression; orange indicates low expression.

Sixteen DEGs were identified in both the CGH and TCGA datasets (Table 4). A heat map of these 16 DEGs in the CGH dataset is presented in Figure 2. As stated previously, gene expression data and age, grade and depth of myometrial invasion, and other clinical information were used to tune the predictive model. Machine learning was performed to select features. One clinical feature (depth of myometrial invasion) and eight DEGs (*ASRGL1, ESR1, EYA2, MSX1, RHEX, SCGB2A1, SOX17*, and *STX18*) were identified. For the CGH dataset, the model achieved 100% sensitivity and 88.9% specificity, whereas for TCGA data, these were 97% sensitivity and 38.7% specificity, respectively. The contingency tables of the agreement between the datasets along with the predictive model are displayed in Table 5. Only one patient with LNM in TCGA (Table 5A) was incorrectly classified; other LNM predictions for both datasets (Tables 5A,B) were correct.

**Table 4:** LN (+) versus LN (–) DEGs in CGH and TCGA datasets.

	**CGH dataset (n** **=** **24)**			**TCGA dataset (n** **=** **289)**		
	**LN (+)**	**LN (–)**	***p***	**LN (+)**	**LN (–)**	***p***
	***n* = 6**	***n* = 18**		***n* = 33**	***n* = 256**	
*SCGB2A1*	−6.48	−1.35	6.54E-04	−1.75	−0.26	5.35E-03
*RHEX*	−9.44	−4.35	5.94E-05	−5.21	−4.48	4.14E-02
*EYA2*	−9.37	−5.30	4.46E-04	−4.25	−3.25	2.74E-02
*MSX1*	−6.96	−3.59	1.18E-02	−3.22	−1.94	6.78E-04
*SOX17*	−7.58	−3.90	4.46E-04	−3.21	−2.39	4.44E-02
*ASRGL1*	−7.56	−3.69	2.97E-05	−4.37	−3.55	8.31E-03
*STX18*	−7.15	−4.09	9.51E-04	−4.73	−3.92	1.17E-03
*ESR1*	−8.49	−5.49	4.43E-03	−5.40	−4.58	8.36E-03
*TMEM101*	−8.07	−5.16	3.37E-03	−5.38	−4.43	1.83E-03
*CFI*	−8.20	−4.94	2.82E-04	−5.32	−4.65	1.81E-02
*LRIG1*	−8.12	−5.62	6.54E-04	−4.85	−4.18	2.82E-02
*ELP3*	−8.03	−5.97	4.46E-04	−5.31	−4.69	1.84E-03
*BASP1*	−7.87	−5.81	7.40E-03	−3.53	−4.14	7.33E-03
*SERINC2*	−5.68	−3.92	5.77E-03	−2.87	−2.13	1.83E-02
*NOXA1*	−7.51	−5.92	4.43E-03	−5.26	−4.47	3.27E-04
*HOXB5*	−7.42	−5.18	1.18E-02	−5.93	−4.83	6.64E-03

*Data are expressed as log_2_ (intensity of corresponding gene/intensity of RPL19)*.

**Table 5:** Contingency table of level of agreement between the two datasets (**A**. TCGA and **B**. CGH) and sequencing-based prediction model.

		**Predicted as**	
		**Positive**	**Negative**
**A**			
**TCGA dataset**	Positive	32	1
	Negative	157	99
**B**			
**CGH dataset**	Positive	6	0
	Negative	2	16

*Patients classified correctly (Pos-Pos and Neg-Neg) are highlighted in yellow. Negative, patients with negative lymph node status; positive, patients with positive lymph node status*.

### PCR Validation and PCR-Based Prediction Model Construction

We used another independent cohort of 72 patients with early stage EEC for qRT-PCR validation of the eight identified DEGs (*ASRGL1, ESR1, EYA2, MSX1, RHEX, SCGB2A1, SOX17*, and *STX18*). Other 17 patients without complete pelvic and para-aortic lymphadenectomy were excluded. The cohort's (*n* = 72) clinical characteristics are listed in Table 3. All 72 patients had clinical T1 tumors (confined to the uterus), with 63 patients (87.5%) being LN (–) and 9 (12.5%) being LN (+). The medians of lymph nodes harvested were 17 in the LN (–) group and 26 in the LN (+) group. Similar to those in TCGA data, these patients with LNM were more likely to have high-grade tumors (*p* = 0.0001) and deep myometrial invasion (*p* = 0.023). The LN (+) patients also had a higher proportion of LVSI (*p* < 0.0001).

**Figure 3:**
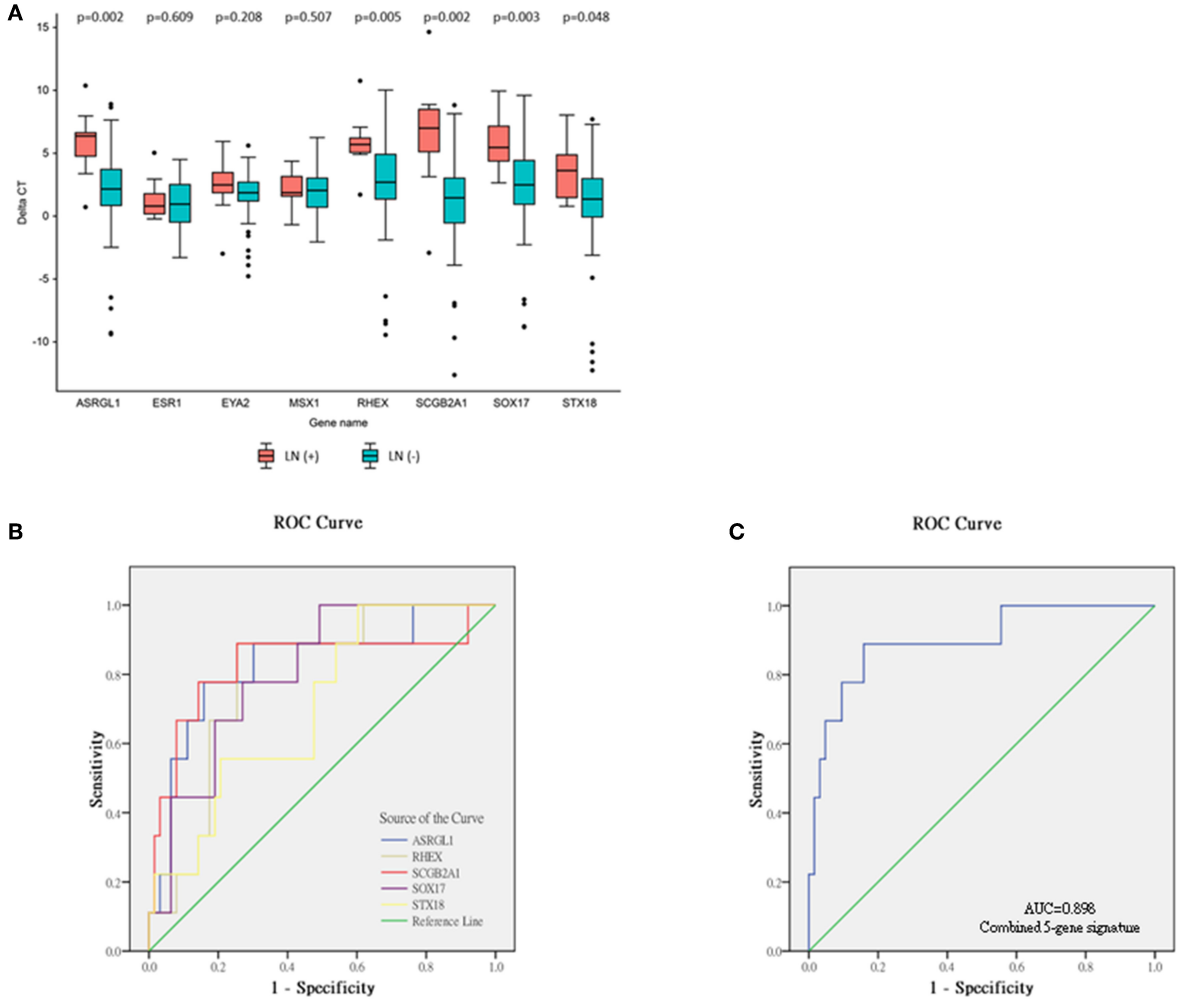
Relative expression levels of eight genes selected from validation cohort, and ROC analyses of single-mRNA/five-mRNA expression. **(A)** Data expressed as (threshold cycle of corresponding gene—threshold cycle of RPL19). A positive delta CT indicates lower expression than that of reference gene *RPL19*. Significant difference determined using the Mann–Whitney *U*-test in five genes—*ASRGL1, RHEX, SCGB2A1, SOX17*, and *STX18*. **(B)** ROC curve of expression of five single mRNAs. **(C)** ROC curve of combined five-mRNA expression.

The relative expression of the eight DEGs is illustrated in Figure 3A. Five DEGs were observed between the LN (+) and LN (–) groups. Significantly lower relative expression of *ASRGL1* (*p* = 0.002), *RHEX* (*p* = 0.005), *SCGB2A1* (*p* = 0.002), *SOX17* (*p* = 0.003), and *STX18* (*p* = 0.048) was discovered in the LN (+) group. After the expression of these five genes and the one clinical parameter (depth of myometrial invasion) were incorporated, the performance of the various machine learning model was determined, as shown in Table 6. The random forest model had the highest sensitivity and specificity: 92 and 31%, respectively. The accuracy, PPV, and NPV of the PCR-based prediction model were 40, 16, and 97%, respectively.

**Table 6:** Prediction performance of PCR-based model using five-gene panel with various machine learning approaches (SMO, sequential minimal optimization).

	**Naive Bayes (%)**	**J48 (%)**	**SMO (%)**	**Random forest (%)**
Accuracy	62	53	36	40
Sensitivity	83	83	89	92
Specificity	61	49	29	31
PPV	23	19	15	16
NPV	96	95	95	97

### Construction of mRNA Signature and Risk Score Formulation

To evaluate the performance of these mRNA markers for predicting LNM in EEC, ROC analysis was performed (Figures 3B,C). The AUC, sensitivity, and specificity of each mRNA marker are listed in Table 7. The AUC of the five mRNAs ranged from 0.705 to 0.829, indicating that all five markers could distinguish LN (+) patients from LN (–) patients (Figure 3B). The sensitivity and specificity of the mRNA markers for detecting patients with LN (+) ranged from 77.8 to 88.9% and 54.0 to 74.6%, respectively. Among the five mRNAs studied, *SCGB2A1* was identified as the best marker for identifying LN (+) patients (AUC = 0.829) because it had the highest sensitivity and specificity.

**Table 7:** Performance of mRNA expression markers in predicting lymph node status in EEC.

**Marker**	**Sensitivity(%)**	**Specificity(%)**	**AUC (95% CI)**	**PPV(%)**	**NPV(%)**
*ASRGL1*	88.9	69.8	0.827 (0.671–0.983)		
*RHEX*	88.9	74.6	0.792 (0.662–0.922)		
*SCGB2A1*	88.9	74.6	0.829 (0.644–1.014)		
*SOX17*	77.8	73.0	0.804 (0.678–0.930)		
*STX18*	88.9	54.0	0.705 (0.544–0.867)		
Combined five mRNA signature	88.9	84.1	0.898 (0.781–1.014)	44.4	98.1

To enable these mRNA signatures to be applied in clinical practice and to improve the mRNA prediction efficacy, for each patient in the validation cohort a risk score was calculated on the basis of the five-mRNA combination. On the basis of the optimal risk score cut-off of −1.77, the 72 patients with EEC in the validation cohort were classified as being at high risk (*n* = 18; risk score > −1.77) or low risk (*n* = 54; risk score < −1.77). The distribution of clinicopathological characteristics in relation to risk score is displayed in Supplement Table 4. The risk score was significantly higher for patients with grade 3 tumors than those with grade 1 or 2 tumors (−3.06, −2.17, and −1.65 for grade 1–3 tumors, respectively, *p* = 0.034). The LN (+) group also had significantly higher risk scores than the LN (–) group (−0.36 vs. −3.08, respectively, *p* < 0.0001). Additionally, the predictive performance of the combined set of five mRNAs was significantly higher than that of any single mRNA marker and achieved an excellent AUC of 0.898, sensitivity of 88.9%, specificity of 84.1%, accuracy of 84.7%, PPV of 44.4%, and NPV of 98.1% (Figure 3C and Table 7).

## Discussion

LNM is a critical prognostic factor for EEC, and the status of lymph nodes is an essential consideration when making decisions about personalized treatment for EEC, especially in patients with the early stage of the disease. Accurately diagnosing LNM before surgical staging remains difficult. A test that can accurately identify LNM in patients with early stage EEC before staging surgery would have substantial clinical value.

Most previous risk models for LNM prediction have been constructed from intraoperative or final pathological findings (38–40). In a multicenter retrospective study, the Korean Gynecologic Oncology Group developed a preoperative model for predicting LNM in endometrial cancer (KGOG-2014) and identified serum CA-125 level plus magnetic resonance imaging as a combination achieving accurate identification of low LNM risk among patients with endometrial cancer (17). However, that study included patients with non-endometrioid histology.

The molecular mechanisms underlying the development, invasion, and metastasis of EEC have not yet been fully characterized and consequently are poorly understood (41). The clinical heterogeneity of patients with EEC probably reflects variation at the molecular level (42); thus, the metastasis mechanisms of EEC tumors likely vary as well.

We employed a genome-wide strategy for characterization of the molecular signatures associated with LNM in early stage EEC; mRNA sequencing data were comprehensively analyzed to identify five mRNAs closely associated with LNM in patients with early stage EEC. Assuming that a combination of several mRNAs has higher sensitivity and specificity than a single mRNA for prediction of LNM, we formulated a risk score on the basis of these five mRNAs. Using the five-mRNA signature, we could effectively categorize patients in our validation cohort as high or low risk, and favorable reliability was achieved in our prediction of LNM in EEC. Logically, our next step is to prospectively validate the use of this gene expression signature in presurgical biopsy specimens of patients with EEC.

The most vital contribution of our study is preoperative LNM risk determination. In most cases, endometrial cancer is diagnosed through endometrial biopsy (43, 44) or fractional dilatation and curettage (43, 45). Once EEC has been diagnosed, presurgical biopsy specimens may be used for molecular testing for our proposed five-gene PCR-based markers. Thus, patients at high risk can be identified before staging surgery. Additionally, low-LNM-risk patients, for whom systemic lymph node dissection may be unnecessary, can be identified. In addition, SLN mapping can be included in prospective studies to reduce surgical morbidities.

Another advantage of our study is its homogeneity in the following respects. First, all patients had pure endometrioid carcinoma, which was confirmed after staging surgery. Second, all patients received complete surgical staging operations, which included para-aortic and pelvic lymph node dissection. Third, gynecologic oncologists performed all treatments to an adequate extent of lymphadenectomy. In the LN (+) and LN (–) groups, the median numbers of lymph nodes removed were 26 and 17, respectively. One study concluded removal of 11 or more pelvic lymph nodes to be adequate (46).

We identified five PCR-based gene markers (*ASRGL1, RHEX, SCGB2A1, SOX17*, and *STX18*) for predicting LNM in patients with early stage EEC. These five markers and their associations with lymph node status in patients with EEC may reveal novel targets for therapy and further investigation of endometrial cancer.

L-asparaginase is an enzyme encoded by the *ASRGL1* gene in humans. Asparagine and glutamine is hydrolyzed by this enzyme into aspartic and glutamic acid, respectively (47). The enzyme has been employed in non-Hodgkin's lymphoma and acute lymphoblastic leukemia treatment (48–50). Fonnes et al. (51) analyzed *ASRGL1* expression by using immunohistochemistry in curettage specimens from 1,144 women with endometrial carcinoma and discovered LNM to be more common in patients with low *ASRGL1* expression than in those with high *ASRGL1* expression. Low *ASRGL1* expression could also predict LNM independently. This finding is consistent with ours. *RHEX*, also known as *C1orf186*, acts as a transduction factor of the EPO–EPOR signaling pathway and promotes erythroid cell differentiation (52). In humans, uterine *RHEX* expression is high, and the abundance of *RHEX* is determined by the stage of a woman's menstrual cycle (53).

*SCGB2A1*, also known as mammaglobin B (*MGB-2*), is a secretoglobin involved in adenocarcinoma development when the tumor originates in organs such as the ovary and endometrium (54, 55). Tassi et al. (54) employed RT-PCR to analyze *MGB-2* expression in EEC tumors with different grades of differentiation and found this gene was expressed in 10 of 11 grade 1 (91%), 16 of 17 in grade 2 (94%), and 6 of 22 grade 3 (27%) EEC cases. Pairwise comparisons of *MGB-2* gene expression levels between the grades 1 or 2 cases and grade 3 cases revealed significantly higher levels in the grades 1 and 2 cases (grade 2 vs. grade 3: *p* < 0.001; grade 1 vs. grade 3: *p* = 0.016). In our series, we discovered that patients with LNM have lower *SCGB2A1*expression levels. This can be attributed to the higher tumor grade of patients with LNM. *SOX17* is a critical antagonist and inhibitor of the Wnt signaling pathway (56). Endometrial tumors expressing higher *SOX17* levels are associated with higher recurrence-free survival rates; conversely, progression of endometrial cancer may be promoted by low *SOX17* expression through downregulation of *MAML3* expression and Wnt signaling (57). A soluble N-ethylmaleimide-sensitive factor attachment protein receptor, *STX18* is involved in several cellular activities, such as the cell cycle and organelle assembly (58). Downregulation of *STX18* was reported as significantly increasing growth of the MCF-7 human breast cancer cells (59).

These reports combined with our present analysis support the appropriateness of using our five-gene signature for predicting LNM in EEC. Nonetheless, our study may have some shortcomings. The primary shortcoming is the small sample size. Only 72 patients were included in the validation cohort. Prior to clinical application of the mRNA signature, larger independent cohorts recruited from multiple centers should be used to confirm the signature's predictive performance. Another shortcoming is that we analyzed only Asian patients. Whether our finding is applicable to non-Asian populations is unknown. Compared with Caucasian populations, other populations with endometrial cancer are at higher risk for LNM and have poorer prognoses (60, 61). Whether ethnic characteristics affect the predictive results is unclear, but we recommend testing our model in non-Asian populations.

In summary, after comprehensive analysis, we established a model with a five-gene expression profile that can serve as a useful tool for predicting LNM in patients with early stage EEC. The predictive model should be tested prospectively and used in conjunction with the present clinical assessments to optimize individual treatment plans for patients with EEC before the first treatment.

## Data Availability Statement

The data used in this study can be obtained from the National Center for Biotechnology Information Sequence Read Archive (bioproject access number: PRJNA554534).

## Ethics Statement

The studies involving human participants were reviewed and approved by institutional review board of Cathay General Hospital (CGH). The patients/participants provided their written informed consent to participate in this study.

## Author Contributions

C-YH, T-HC, and H-DH designed the study and provided the ideas. C-YH, T-HC, K-WL, and H-DH designed the experiments. C-YH and S-HH provided materials. SS, C-DY, M-YC, H-TH, and H-CH conducted the experiments. C-YH, C-HC, and T-HC performed the analysis and created and adjusted the figures and tables. C-YH and T-HC wrote the manuscript. All authors read and approved the final manuscript.

### Conflict of Interest

The authors declare that the research was conducted in the absence of any commercial or financial relationships that could be construed as a potential conflict of interest.
