# Supplementary material for: Pilot Study to Establish a Novel Five-Gene Biomarker Panel for Predicting Lymph Node Metastasis in Patients With Early Stage Endometrial Cancer
Source: Front Oncol. 2020 Jan 21;9:1508. doi: 10.3389/fonc.2019.01508 (PMC6985442; doi:10.3389/fonc.2019.01508)
Supplement: Supplementary file 1 [file Data_Sheet_1.docx]

**Supplementary Materials**

**Supplement Table 1. Reporting recommendations for tumor marker prognostic studies (REMARK) guidelines.**

| **Item to be reported** | |
| --- | --- |
| **INTRODUCTION** | |
| 1 | *State the marker examined, the study objectives, and any pre-specified hypotheses.*  A multistep case-control study was designed to identify predictive mRNA markers for lymph node metastasis in clinically early stage EEC patients. Figure 1 presents a study workflow schematic.  1) In biomarker screening phase, RNA sequencing data were analyzed in CGH dataset and TCGA dataset to identify differentially expressed genes between LN (+) group and LN (-) group.  2) In biomarker selection phase, machine learning was used to establish sequence-based prediction model.  3) In biomarker validation phase, real-time quantitative PCR (RTQ-PCR) was used to validate the predictive performance of the 8 biomarkers identified by machine learning.  Examined markers:  Study cohort: *ASRGL1*, *RHEX*, *ESR1*, *EYA2*, *MSX1*, *SCGB2A1*, *SOX17*, and *STX18* (Supplement Table 3).  Study objectives: to establish a gene biomarker for prediction of LNM in early stage EEC. |
| **MATERIALS AND METHODS** | |
| *Patients* | |
| 2 | *Describe the characteristics (e.g., disease stage or co-morbidities) of the study patients, including their source and inclusion and exclusion criteria.*  The clinicopathological characteristics, source, inclusion and exclusion criteria of the EEC patients of this study are described in the Methods section 1.1 (“Patient Enrollment and Sampling”), Table 1, Table 2, and Table 3. |
| 3 | *Describe treatments received and how chosen (e.g., randomized or rule-based).*  Treatment protocols were based on NCCN treatment guidelines. Only patients underwent total hysterectomy and/or bilateral salpingo-oophorectomy, bilateral pelvic lymph node dissection, and para-aortic lymphadenectomy by laparotomy or laparoscopy were included. |
| *Specimen characteristics* | |
| 4 | *Describe type of biological material used (including control samples) and methods of preservation and storage.*  Tissue specimens collected after the hysterectomies were immediately preserved in RNAlater® (Qiagen, Valencia, CA, USA) solution and stored at -80°C until analysis. The analyses were performed by the department of obstetrics and gynecology at Cathay General Hospital (Taipei, Taiwan) for clinically early-stage (stage I) endometrial endometrioid carcinoma patients, diagnosed between March 2011 to December 2017. |
| *Assay methods* | |
| 5 | *Specify the assay method used and provide (or reference) a detailed protocol, including specific reagents or kits used, quality control procedures, reproducibility assessments, quantitation methods, and scoring and reporting protocols. Specify whether and how assays were performed blinded to the study endpoint.*  As shown in figure 1, we performed RNA sequencing for biomarker screening in 24 out of 113 enrolled patients. The detailed protocol is as described in “RNA Sequencing and Data Processing” in the methods section 1.2. The laboratory staff members were all blinded to the study endpoint: lymph node status.  In the biomarker validation phase, the mRNA expression levels of eight genes, namely, *ASRGL1*, *ESR1*, *EYA2*, *MSX1*, *RHEX*, *SCGB2A1*, *SOX17*, and *STX18*, were examined by TaqMan real-time quantitative (RTQ) RT-PCR in triplicate and compared with expression level of *RPL19* in 72 out of 113 patients. The detailed protocol was described “qRT-PCR Validation and PCR-Based Prediction Model Construction Through Machine Learning” in the Methods section 4. |
| *Study design* | |
| 6 | *State the method of case selection, including whether prospective or retrospective and whether stratification or matching (e.g., by stage of disease or age) was used. Specify the time period from which cases were taken, the end of the follow-up period, and the median follow-up time.*  The endometrial endometrioid carcinoma patients (n=113) were retrospectively selected from the departments of obstetrics and gynecology at Cathay General Hospital in northern Taiwan. The patients were diagnosed between March 2011 and December 2017. Clinicopathological characteristics including the information of lymph node status were obtained after staging operation (laparotomy or laparoscopy) for EEC. The end of the follow-up-period was December 31, 2018 and the median follow-up time was 36 months. |
| 7 | *Precisely define all clinical endpoints examined.*  The clinical endpoint was the lymph node status of EEC patients. Lymph node status was considered positive if metastasis was histologically proven and negative if no metastasis was detected in the surgical specimen and during at least the subsequent 1 year. |
| 8 | *List all candidate variables initially examined or considered for inclusion in models.*  The overlapping differentially expressed genes (DEGs) in the CGH dataset and TCGA dataset were selected as core DEGs. In addition to gene expression data, clinical information, such as age, tumor grade, and depth of myometrial invasion, were also used for prediction model tuning. The details were described in “Sequencing-Based Model Construction and Feature Selection Through Machine Learning” in the Methods section 3. |
| 9 | *Give rationale for sample size; if the study was designed to detect a specified effect size, give the target power and effect size.*  This is a pilot study. 24 samples were used for RNA sequencing for biomarker screening. In the biomarker validation phase, all available clinical samples including a total of 72 clinically early stage EEC tumor samples were used for biomarker validation. Another 17 tumor samples without complete pelvic and para-aortic lymphadenectomy were not used. |
| *Statistical analysis methods* | |
| 10 | *Specify all statistical methods, including details of any variable selection procedures and other model-building issues, how model assumptions were verified, and how missing data were handled.*   1. *Study cohort selection*   “Seq-Based Model Construction and Feature Selection” in the Results section describes the selection of the 16 promising DEGs as biomarkers in the CGH dataset and the TCGA dataset (Table 4)   1. *Statistical methods*   Binary logistic regression analysis was used to generate a set of multi-mRNA markers (combination of five mRNA markers) to predict the lymph node status. We then devised a risk score – a linear combination of the expression levels of these five mRNAs. The details were as described in “Risk Score Calculation and Predictive Model Construction” in the Methods section 5.   1. *Association of risk score with clinicopathological characteristics*   The distribution of clinicopathological characteristics in relation to risk score is shown in Supplement Table 4.   1. *Missing data*   In the validation cohort, patients with missing values were excluded. |
| 11 | *Clarify how marker values were handled in the analyses; if relevant, describe methods used for cutpoint determination.*  The risk score of lymph node metastases for each patient was calculated as follows: Risk score = 0.249 × (Ct of *ASRGL1* − Ct of *RPL19*) + 0.166 × (Ct of *RHEX* − Ct of *RPL19*) + 0.434 × (Ct of *SCGB2A1* − Ct of *RPL19*) + 0.02 × (Ct of *SOX17* − Ct of *RPL19*) − 0.51 × (Ct of *STX18* − Ct of *RPL19*) − 4.06, where Ct is the threshold cycle of each gene. The cutpoint was determined as the value whose sensitivity and specificity are the closest to the value of the area under the ROC curve and the absolute value of the difference between the sensitivity and specificity values is minimum. The details were described in “Risk Score Calculation and Predictive Model Construction” in the Methods section 5. |
| **RESULTS** | |
| *Data* | |
| 12 | *Describe the flow of patients through the study, including the number of patients included in each stage of the analysis (a diagram may be helpful) and reasons for dropout. Specifically, both overall and for each subgroup extensively examined report the numbers of patients and the number of events.*  The flow of patients through the study was shown in Figure 1. The details of the EEC patients used in the different analyses are reported in Table 1, 2, and 3. A total of 113 clinically early stage EEC patients were enrolled.  1) 24 samples were used for RNA sequencing for biomarker screening.  2) In the biomarker validation phase, all available clinical samples with a total of 72 clinically early stage EEC tumor samples were used for biomarker validation.  3) Another 17 tumor samples without complete pelvic and para-aortic lymphadenectomy were not used. |
| 13 | *Report distributions of basic demographic characteristics (at least age and sex), standard (disease-specific) prognostic variables, and tumour marker, including numbers of missing values.*  The clinicopathological characteristics of 24 patients used for RNA sequencing experiment and 72 patients used for RTQ-PCR experiment were shown in Table 1 and 3. The distribution of clinicopathological characteristics in relation to risk score was shown in Supplement Table 4. |
| *Analysis and presentation* | |
| 14 | *Show the relation of the marker to standard prognostic variables.*  The distribution of clinicopathological characteristics in relation to risk score was shown in Supplement Table 4. |
| 15 | *Present univariable analyses showing the relation between the marker and outcome, with the estimated effect (e.g., hazard ratio and survival probability). Preferably provide similar analyses for all other variables being analysed. For the effect of a tumour marker on a time-to-event outcome, a Kaplan-Meier plot is recommended.*  Not applicable because the primary endpoint of this study is to predict the lymph node status. |
| 16 | *For key multivariable analyses, report estimated effects (e.g., hazard ratio) with confidence intervals for the marker and, at least for the final model, all other variables in the model.*  Not applicable. The sensitivity, specificity, PPV, NPV, and AUC of each gene and combined use of mRNAs are listed in Table 7. |
| 17 | *Among reported results, provide estimated effects with confidence intervals from an analysis in which the marker and standard prognostic variables are included, regardless of their statistical significance.*  Not applicable because the primary endpoint of this study is to predict the lymph node status. |
| 18 | *If done, report results of further investigations, such as checking assumptions, sensitivity analyses, and internal validation.*  The results of the validation cohort are specified in the Results section. |
| **DISCUSSION** | |
| 19 | *Interpret the results in the context of the pre-specified hypotheses and other relevant studies; include a discussion of limitations of the study.*  The study results were interpreted in the context of pre-specified hypotheses and other relevant studies in the Discussion section. The limitations of this study were also discussed in the Discussion section. |
| 20 | *Discuss implications for future research and clinical value.*  Further investigation using larger patient cohorts, or *in vitro* and *in vivo* models could further validate the clinical and biological significance of the examined biomarkers in this study. |

**Supplement Table 2. Primers used in qRT-PCR.**

| **Gene Name** | **Upstream Primer (5'-3')** | **Downstream Primer (5'-3')** |
| --- | --- | --- |
| ***ASRGL1*** | GCTGGAGGTTATGCCGACAATG | ATAGGTCCGCAGCCTCTTCTAC |
| ***ESR1*** | GCTTACTGACCAACCTGGCAGA | GGATCTCTAGCCAGGCACATTC |
| ***EYA2*** | TCCTCCAGGAGGCATCTCACAA | TCTGTGTCTCCCTCTTTCGCTG |
| ***MSX1*** | GACTCCTCAAGCTGCCAGAAGA | ACGGTTCGTCTTGTGTTTGCGG |
| ***RHEX*** | TAGCTTGGATAGCTCCTGCAGTTC | CATTTTTTAGTTCTCCAGGGTCAGA |
| ***SCGB2A1*** | GCAGTGTTTCCTCAACCAGTCAC | GCCATAGTCTGTAGCCCTCTGA |
| ***SOX17*** | ACGCTTTCATGGTGTGGGCTAAG | GTCAGCGCCTTCCACGACTTG |
| ***STX18*** | GGAGGATGACAGACACAGAACG | GCTCCTTCACTTGCTGGGAATG |
| ***RPL19 (reference gene)*** | TCACAGCCTGTACCTGAAGGTG | CGTGCTTCCTTGGTCTTAGACC |

**Supplement Table 3. Alignment and mapping quality metrics produced by Qualimap over the output BAM files from alignment with STAR.**

| **Sample ID** | **Raw Data** | **QC** | **Align** | **QC/RawData** | **Align/QC** | **Align/RawData** |
| --- | --- | --- | --- | --- | --- | --- |
| end_11 | 19,087,998 | 18,537,593 | 17,696,021 | 97.12% | 95.46% | 92.71% |
| end_13 | 16,629,680 | 16,132,571 | 15,250,050 | 97.01% | 94.53% | 91.70% |
| end_15 | 21,100,563 | 20,592,224 | 19,743,500 | 97.59% | 95.88% | 93.57% |
| end_17 | 22,167,599 | 21,614,916 | 20,760,419 | 97.51% | 96.05% | 93.65% |
| end_19 | 21,367,417 | 20,850,556 | 20,050,778 | 97.58% | 96.16% | 93.84% |
| end_22 | 22,599,700 | 22,053,784 | 21,223,126 | 97.58% | 96.23% | 93.91% |
| end_24 | 20,182,896 | 19,670,398 | 18,943,648 | 97.46% | 96.31% | 93.86% |
| end_25 | 19,232,062 | 18,754,998 | 18,029,322 | 97.52% | 96.13% | 93.75% |
| end_26 | 20,679,552 | 20,156,727 | 19,352,959 | 97.47% | 96.01% | 93.59% |
| end_31 | 20,616,572 | 20,131,050 | 19,356,892 | 97.65% | 96.15% | 93.89% |
| end_32 | 17,710,127 | 17,245,830 | 16,556,447 | 97.38% | 96.00% | 93.49% |
| end_37 | 19,624,604 | 19,135,290 | 18,393,174 | 97.51% | 96.12% | 93.73% |
| end_3 | 19,357,107 | 18,674,618 | 17,756,010 | 96.47% | 95.08% | 91.73% |
| end_45 | 21,494,022 | 20,933,229 | 20,138,880 | 97.39% | 96.21% | 93.70% |
| end_46 | 21,882,437 | 21,323,806 | 20,495,696 | 97.45% | 96.12% | 93.66% |
| end_50 | 22,879,598 | 22,318,752 | 21,226,609 | 97.55% | 95.11% | 92.78% |
| end_53 | 22,565,893 | 22,015,402 | 20,877,436 | 97.56% | 94.83% | 92.52% |
| end_54 | 22,724,961 | 22,174,931 | 21,176,597 | 97.58% | 95.50% | 93.19% |
| end_58 | 19,135,981 | 18,646,519 | 17,926,448 | 97.44% | 96.14% | 93.68% |
| end_59 | 23,139,971 | 22,566,890 | 21,732,952 | 97.52% | 96.30% | 93.92% |
| end_60 | 22,522,175 | 21,994,982 | 21,148,415 | 97.66% | 96.15% | 93.90% |
| end_61 | 21,985,548 | 21,445,457 | 20,553,144 | 97.54% | 95.84% | 93.48% |
| end_62 | 21,447,812 | 20,906,310 | 20,089,918 | 97.48% | 96.10% | 93.67% |
| end_8 | 19,528,005 | 19,017,744 | 18,232,031 | 97.39% | 95.87% | 93.36% |

|  | **n** | **Risk Score** | ***p*** |
| --- | --- | --- | --- |
| **Age, years**  < 55  > 55 | 36  36 | -2.96  -2.53 | 0.281 |
| **Tumor grade**  1 (n=51)  2 (n=12)  3 (n=9) | 51  12  9 | -3.06  -2.17  -1.65 | **0.034** |
| **Myometrial invasion**  Inner 1/2  Outer 1/2 | 44  28 | -2.92  -2.46 | 0.271 |
| **Lymph node status**  Negative  Positive | 63  9 | -3.08  -0.36 | **< 0.0001** |

**Supplement Table 4. Distribution of clinicopathological characteristics in relation to risk score in the validation cohort.**
